# Surface engineering of lipid nanoparticles: targeted nucleic acid delivery and beyond

**DOI:** 10.52601/bpr.2023.230022

**Published:** 2023-10-31

**Authors:** Yi Lin, Qiang Cheng, Tuo Wei

**Affiliations:** 1 State Key Laboratory of Stem Cell and Reproductive Biology, Institute of Zoology, Chinese Academy of Sciences, Beijing 100101, China; 2 Department of Biomedical Engineering, College of Future Technology, Peking University, Beijing 100871, China; 3 Beijing Institute for Stem Cell and Regenerative Medicine, Beijing 100101, China; 4 University of Chinese Academy of Sciences, Beijing 100049, China

**Keywords:** Lipid nanoparticles, Nucleic acid delivery, Surface engineering, Targeted delivery

## Abstract

Harnessing surface engineering strategies to functionalize nucleic acid-lipid nanoparticles (LNPs) for improved performance has been a hot research topic since the approval of the first siRNA drug, patisiran, and two mRNA-based COVID-19 vaccines, BNT162b2 and mRNA-1273. Currently, efforts have been mainly made to construct targeted LNPs for organ- or cell-type-specific delivery of nucleic acid drugs by conjugation with various types of ligands. In this review, we describe the surface engineering strategies for nucleic acid-LNPs, considering ligand types, conjugation chemistries, and incorporation methods. We then outline the general purification and characterization techniques that are frequently used following the engineering step and emphasize the specific techniques for certain types of ligands. Next, we comprehensively summarize the currently accessible organs and cell types, as well as the other applications of the engineered LNPs. Finally, we provide considerations for formulating targeted LNPs and discuss the challenges of successfully translating the “proof of concept” from the laboratory into the clinic. We believe that addressing these challenges could accelerate the development of surface-engineered LNPs for targeted nucleic acid delivery and beyond.

## INTRODUCTION

Nucleic acids are natural biomacromolecules that were first discovered in the nucleus of eukaryotic cells and are now known to be found in all living organisms (Sessler *et al.* 2007). Since the demonstration of their molecular structure and role in genetic inheritance, the understanding of nucleic acid chemistry and biology has continued to grow (Bao and Suresh 2003). Deoxyribonucleic acid (DNA) and ribonucleic acid (RNA) are two main classes of nucleic acids that carry genetic information. The central dogma of molecular biology describes the general transfer direction of sequence information: DNA encodes genetic information called genes and is transcribed to messenger RNA (mRNA); mRNA delivers information from genes to ribosomes and directs the synthesis of proteins (Crick 1970). This normal information flow in biological systems reveals that either nucleic acid-encoded variations or abnormal patterns of gene expression can cause genetic disorders and be associated with disease development. Conventional drugs, such as small molecules and antibodies, are developed to directly target certain proteins produced at the end of the process (Hoelder *et al.* 2012). However, small-molecule drugs work by binding to the structural pockets of proteins, and those proteins whose functional interfaces lack such pockets are often considered “undruggable” (Henley and Koehler 2021). Nucleic acid drugs as novel therapeutic modalities can overcome this limitation, since most of them act in different ways (not to bind the pockets of proteins) and are able to modulate the expression process at various levels (Lächelt and Wagner 2015). For instance, plasmid DNA and mRNA that carry sequence information are constructed and introduced into cells for the substitution of defected genes and production of functional proteins (Ibraheem *et al.* 2014); small interfering RNA (siRNA) and microRNA (miRNA) that target mRNA are designed to produce a gene silencing effect when exposed to the cytosol of cells (Lam *et al.* 2015); and single guide RNA (sgRNA) combined with Cas9 (clustered regularly interspaced short palindromic repeat/CRISPR-associated protein 9) mRNA or protein can be co-administered with or without single stranded DNA (ssDNA) to cells for the precise correction or knockout of disease-causing genes at the genomic level (Lin *et al.* 2022).

Despite their great capacity for modulating gene expression, the central problem that limits widespread applications of nucleic acid drugs in clinics is delivery. Nucleic acid drugs need to overcome several barriers to reach target cells and enter the cytosolic compartment to perform their functions. However, naked nucleic acids are susceptible to nuclease degradation in biological fluids, and they cannot diffuse across cellular membranes due to their high molecular weight as well as their hydrophilic and highly negatively charged nature (Yin *et al.* 2014). Therefore, to fully realize the therapeutic potential of nucleic acid drugs, the delivery problem must be solved. Virus-based vectors represent one of the most efficient carriers for nucleic acid delivery, but they also exhibit significant limitations, including high immunogenicity, low cargo capacity, and complexity of manufacturing (Bulcha *et al.* 2021). In comparison, non-viral vectors based on synthetic materials, such as polymers, lipids, peptides, and inorganic silica materials, are thought to be less immunogenic, and their features like biodegradability, chemical versatility, and ease of scaling up, continue to attract more and more researchers to develop potent and safe carriers for nucleic acid delivery (Yin *et al.* 2014; Kubiatowicz *et al.* 2022).

Among these non-viral delivery systems, lipid nanoparticles (LNPs) are recognized as the most clinically advanced nucleic acid delivery technology since they were used in the first FDA (the US Food and Drug Administration)-approved siRNA drug, patisiran, for the treatment of hereditary transthyretin-mediated amyloidosis (hATTR) and, most recently, were utilized to rapidly develop Pfizer-BioNTech and Moderna’s COVID-19 mRNA vaccines (BNT162b2 and mRNA-1273) against SARS-CoV-2 (Xiao and Shi 2021; Tian *et al.* 2023). LNPs are lipid-based sphere-shape vesicles with a diameter of 50–200 nm that are commonly composed of ionizable lipids, phospholipids, cholesterol, and polyethylene glycol (PEG)-lipids (Zong *et al.* 2023). While tremendous efforts, such as developing novel lipid molecules (Liu *et al.* 2021) and optimizing internal ratios of lipid components (Cheng *et al.* 2018), have been made to improve the potency of LNPs, achieving efficient nucleic acid delivery to specific organs or cell types still presents a critical challenge (Dilliard and Siegwart 2023; Kon *et al.* 2023; Veiga *et al.* 2023; Wei *et al.* 2022). To date, several strategies for fabricating targeted LNPs have been developed, and most of them are based on surface engineering (Belhadj *et al.* 2023; Liang *et al.* 2015; Lin *et al.* 2023; Kasiewicz *et al.* 2023; Kedmi *et al.* 2018; Rurik *et al.* 2022). In this review, we focus on the surface-engineered LNPs for nucleic acid delivery. We review the strategies that have been utilized to decorate the surface of LNPs, summarize the purification and characterization techniques, and discuss the purposes of surface engineering. We also point out the current challenges in this fast-growing field, as we believe that addressing these challenges could accelerate the development of surface-engineered LNPs for targeted nucleic acid delivery and beyond.

## SURFACE ENGINEERING STRATEGIES

LNPs are usually formulated by the solvent dilution method, in which lipid components are first dissolved in a water-miscible solvent (typically ethanol) and then diluted by an acidic buffer (pH 3–4) containing nucleic acids (Wang *et al.* 2023c). The tertiary amines of ionizable lipids are protonated in acidic conditions, which enables the encapsulation of negatively charged nucleic acids by electrostatic interactions (Ferhan *et al.* 2022). Spontaneously, lipid molecules aggregate to bury their hydrophobic lipid tails in the interior and expose their hydrophilic segments to the surface of the formed small particles (Eygeris *et al.* 2022). Next, the buffer exchange is performed to replace acidic buffers with physiological buffers (pH 7.4). The neutralization of ionizable lipids drives the fusion of small particles to form larger particles, and the further fusion process is inhibited by the phase separation of PEG-lipids (Kulkarni *et al.* 2018). It was reported that PEG-lipids and phospholipids together formed the surface layer of LNPs, and ionizable lipids would also be incorporated if LNPs did not contain sufficient phospholipids to cover the exterior monolayer (Kulkarni *et al.* 2018). In addition, PEG also induces the formation of the hydrated layer of LNPs in solution, which further prevents LNPs from aggregation and provides colloidal stability (Cui *et al.* 2022). Besides, the apparent acid-dissociation constant (pKa) of the formed LNPs is below seven, which provides a nearly neutral surface charge under physiological pH conditions (Carrasco *et al.* 2021).

A sheddable PEG-lipid, typically PEGylated dimyristoyl lipid, is frequently used in the aforementioned process. Upon systemic administration, the shedding of PEG-lipids breaks the equilibrium of surface lipid compositions, which promotes the adsorption of serum proteins to the surface of LNPs (Dilliard *et al.* 2021). The adsorbed proteins are expected to possibly influence organ tropism of LNPs (Cheng *et al.* 2020; Dilliard *et al.* 2021), and this assumption was evidenced by the finding that the adsorption of apolipoprotein E (ApoE) facilitated hepatic entry of LNPs *via* the low-density lipoprotein receptor (LDLR) on hepatocytes in the liver (Akinc *et al.* 2010).

**Figure 1:**
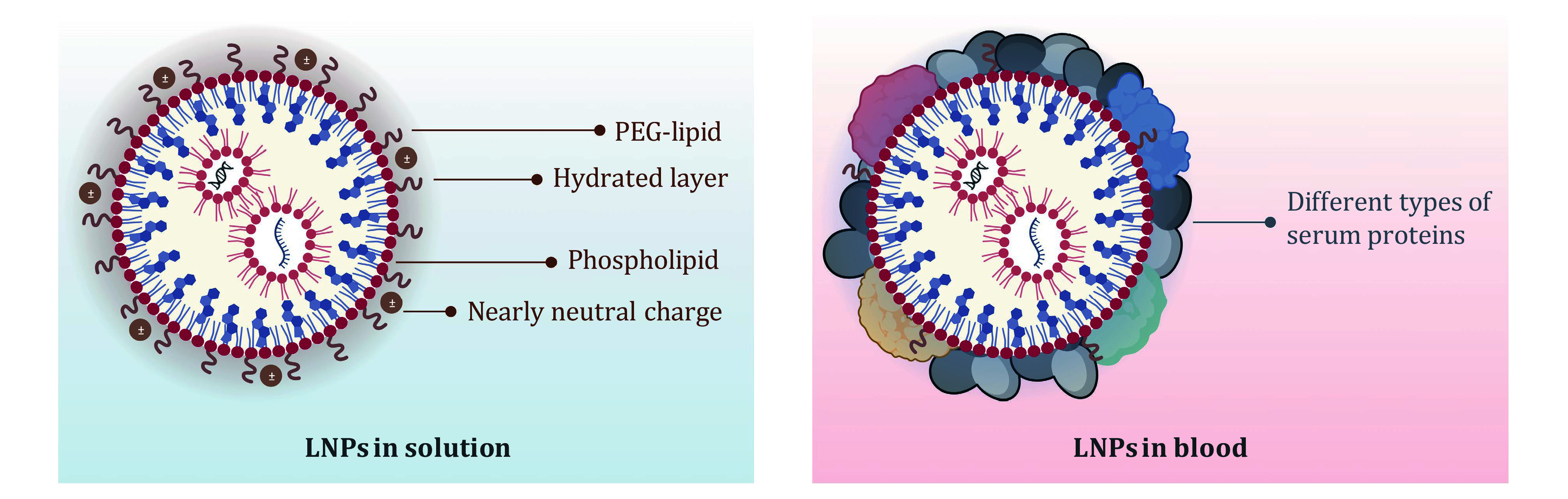
Different surface states of nucleic acid-LNPs in solution and in blood. Created with BioRender.com

Based on the different surface states of LNPs mentioned above (Fig. 1), the surface engineering strategies can be classified into three main categories: (1) substitution of PEGylated dimyristoyl lipid; (2) alteration of surface charge; and (3) modification with targeting ligand. Furthermore, these strategies can be achieved either by direct incorporation of pre-synthesized molecules or by *in situ* conjugation of targeting moieties. Specific chemicals and preparation methods are summarized in the following sections.

### Incorporation of pre-synthesized ligands

**Figure 2:**
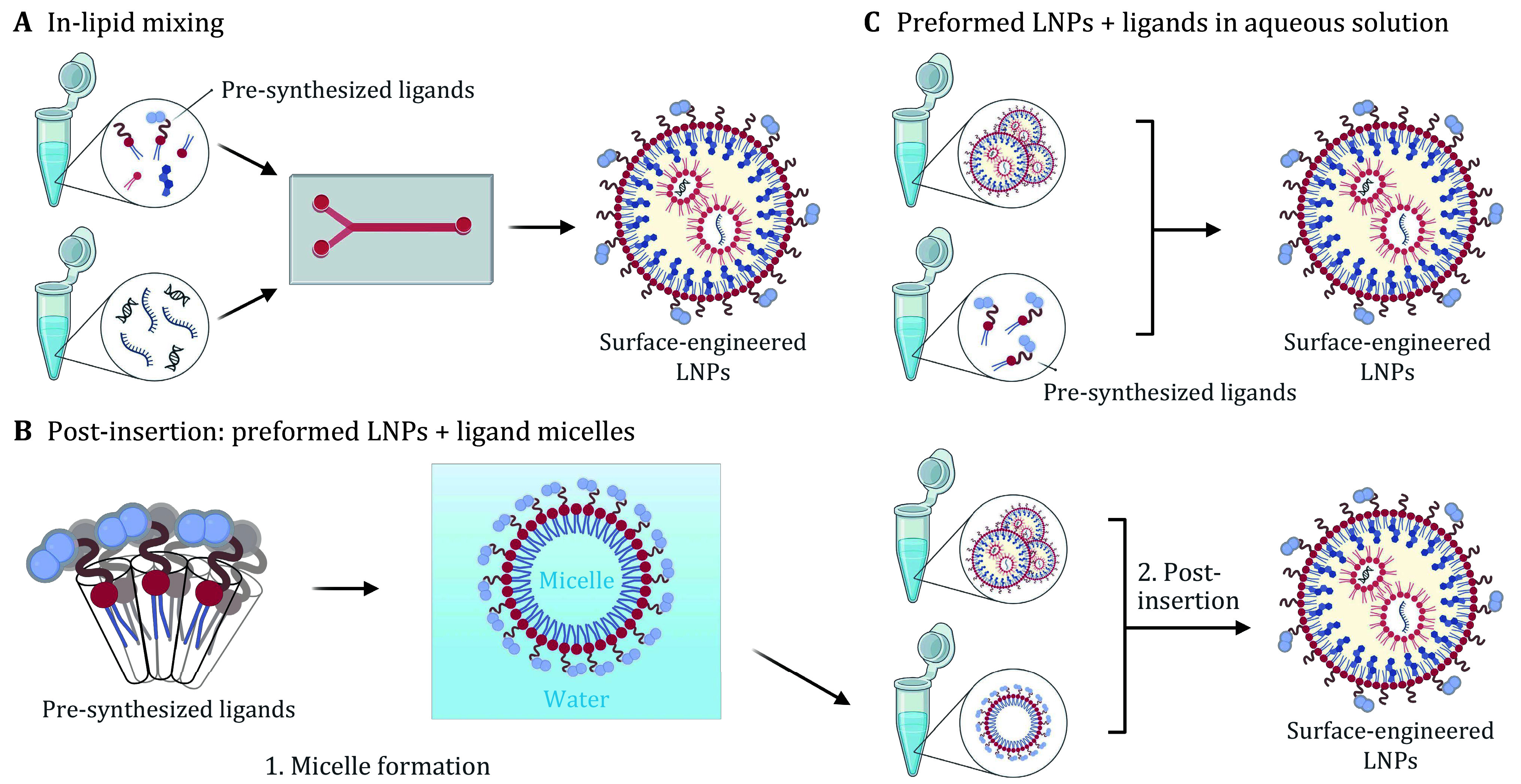
Incorporation methods of pre-synthesized ligands for generating surface-engineered LNPs. **A** Pre-synthesized ligands are dissolved in ethanol with lipid mixtures, and surface-engineered LNPs are prepared by the direct “in-lipid mixing” method. **B** Micelles containing pre-synthesized ligands and LNPs are first formed separately, and surface-engineered LNPs are prepared by the post-insertion method. **C** Pre-synthesized ligands are dissolved in aqueous solution, and surface-engineered LNPs are formed by incubation of preformed LNPs with the ligands. Created with BioRender.com

Lipid molecules, small molecule-lipid conjugates, and peptide-lipid conjugates have defined chemical structures; they are often pre-synthesized and then incorporated into LNPs. Among different incorporation methods, the “in-lipid mixing” method is the most convenient (Fig. 2A). Using this method, additional conjugation chemistry is bypassed, which simplifies the formulation process and requires no further purification. Most pre-synthesized molecules are directly dissolved in lipid mixtures to prepare surface-modified LNPs by the standard ethanol dilution method. For example, polysarcosine-lipids that have shown great stealth properties were synthesized as a PEG-lipid alternative and utilized to fabricate LNPs with a polysarcosine shell on the surface (Nogueira *et al.* 2020; Wilhelmy *et al.* 2023). Small molecules (*e.g.*, mannose and *N*-acetylgalactosamine, GalNAc) and short peptides (*e.g.*, pPB and A5G33 peptides) with targeting ability are also pre-conjugated with PEG-lipids and used to produce targeted LNPs *via* the same method (Jin *et al.* 2023; Kasiewicz *et al.* 2023; Li *et al.* 2018; Zhang *et al.* 2020). Notably, PEGylated distearyl lipids instead of dimyristoyl lipids are often used in this case since the desorption rate of distearyl lipids in the serum is lower than dimyristoyl lipids (Wilson *et al.* 2015; Zhu *et al.* 2017), which ensures that targeting moieties can be present on the surface of LNPs to the utmost extent upon intravenous injection. In addition to the PEG-lipid conjugates, targeting ligands can also be conjugated with other lipid components, such as ionizable lipids (Han *et al.* 2023; Xue *et al.* 2022) and cholesterol (Goswami *et al.* 2019, 2021; Zhang *et al.* 2022), for formulating surface-engineered LNPs. For instance, alendronate, a small molecule that targets the bone component hydroxyapatite, was coupled to the ionizable lipid 480-C14 for generating bone-targeted 490BP-C14 LNPs (Xue *et al.* 2022), and a peptide (NNGGGGCSERSMNFC)-cholesterol conjugate, which targets the intercellular adhesion molecule 1 (ICAM-1) receptor, was synthesized and incorporated to develop airway epithelial cell-specific LNPs (Zhang *et al.* 2022). In addition to these ligand-tethered lipids, differently charged lipids can also be incorporated by the “in-lipid mixing” method. We previously reported a strategy termed Selective ORgan Targeting (SORT) for tissue-specific mRNA delivery (Cheng *et al.* 2020). SORT LNPs were prepared by the addition of charged SORT molecules to conventional four-component LNPs, and it was found that permanently cationic lipids and anionic phospholipids could direct LNPs to the lung and the spleen, respectively.

Besides the “in-lipid mixing” method, pre-synthesized ligands can be inserted into the formed LNPs by the post-insertion technique as well (Fig. 2B). The post-insertion technique was initially developed to produce sterically stabilized immunoliposomal drugs (*e.g.*, Caelyx/Doxil) for increasing their therapeutic index in the treatment of tumors (Ishida *et al.* 1999). The method described that ligand-PEG-DSPE (distearoylphosphatidylethanolamine) could be transferred from a micellar phase into the outer monolayer of preformed liposomes in a time- and temperature-dependent manner (Ishida *et al.* 1999). Owing to its simple, flexible, and effective preparation process, the post-insertion method is widely adopted for the production of targeted lipid-based vesicles, including LNPs. For example, micelles consisting of αvβ3 integrin-targeted cyclic RGD-PEG-DSPE (Sakurai *et al.* 2016) or epithelial cell adhesion molecule (EpCAM)-targeted Epi-1 peptide-PEG-DSPE (Sakurai *et al.* 2017) were synthesized and used to prepare targeted LNPs for treating metastatic lung tumors or Hep-3B hepatocellular cancer, respectively. In a more recent study, the optimized multi-valent GalNAc targeting ligand GL6 was rationally designed and utilized to fabricate the asialoglycoprotein receptor (ASGPR)-targeted LNPs for the delivery of the CRISPR base editing drug to the liver of patients lacking sufficient LDLR activity (Kasiewicz *et al.* 2023). Moreover, the post-insertion method is preferred for the incorporation of long peptide- and polyanionic macromolecule-based ligands, since the long peptide (*e.g.*, RVG peptide) usually contains multiple hydrophobic amino acids that may interfere with the self-assembly process of LNPs, and the polyanionic macromolecule, such as the CH6 aptamer (Liang *et al.* 2015), is highly negatively charged and would be entrapped in the inner core of LNPs, thereby losing the possibility of being displayed on the surface.

Apart from the traditional post-insertion method, pre-synthesized ligands can also be dissolved in aqueous solutions and then directly mixed with preformed LNPs, bypassing the micelle formation step (Fig. 2C). The prerequisite for using this method is that the ligands should be soluble in water and not precipitate during the modification process. For instance, an airway epithelial cell-targeted cleavable peptide Y (cY) that consists of a hydrophilic and positively charged K16 (16 lysines) nucleic acid binding domain was synthesized and mixed with the preformed LNPs in MES buffer to construct peptide-modified LNPs (Sanghani *et al.* 2021). It was speculated that the insertion of cY peptides was driven by the electrostatic interactions between the positively charged K16 domain and negatively charged siRNA deposited in the interior of LNPs.

### *In situ* conjugation of targeting moieties

**Figure 3:**
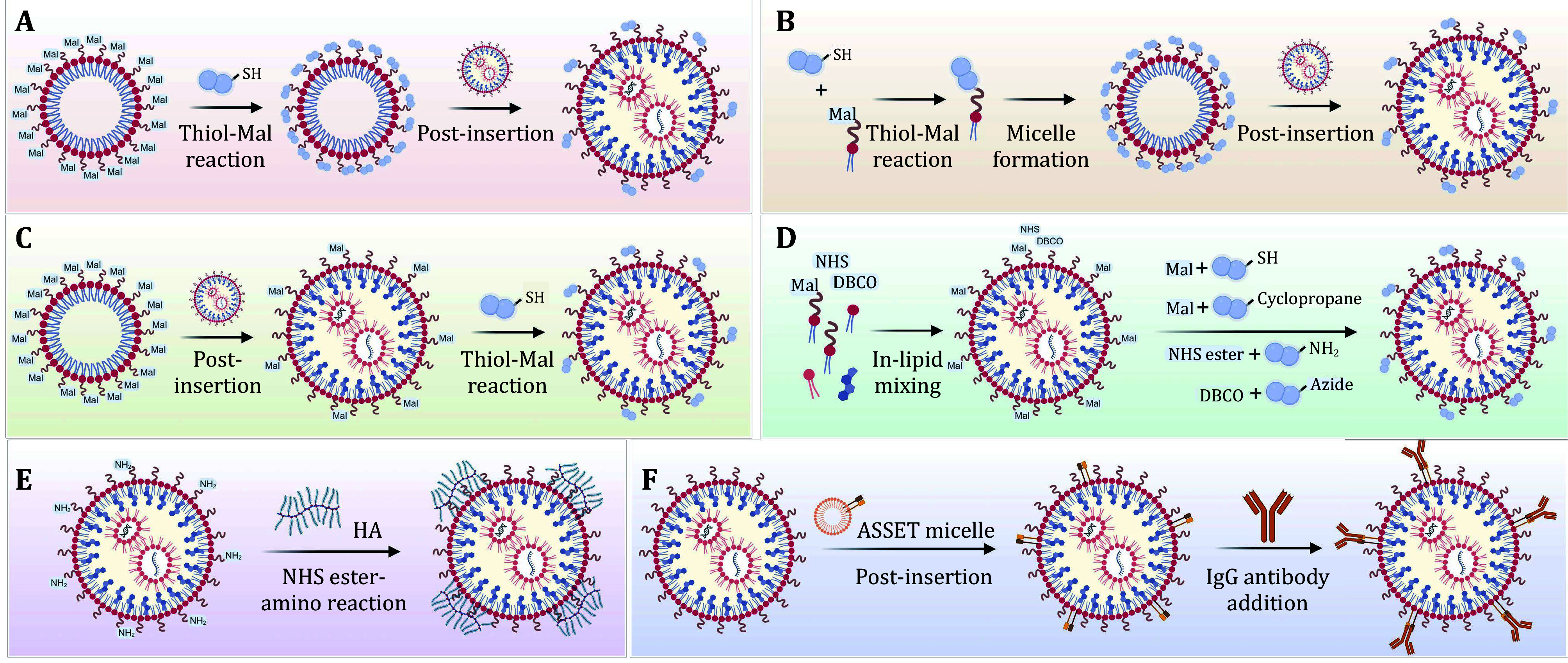
*In situ* modification strategies for generating surface-engineered LNPs. **A** Ligand-micelles are first formed by incubation of preformed Mal-micelles with ligands containing thiol groups, and surface-engineered LNPs are prepared by the post insertion method. **B** Ligand-PEG-lipids are first synthesized by Thiol-Mal reaction, and then ligand-micelles are formed; surface-engineered LNPs are finally prepared by the post insertion method. **C** Mal-LNPs are first formed from Mal-micelles by the post-insertion method, and surface-engineered LNPs are prepared through Thiol-Mal reaction. **D** Functionalized LNPs are formed by the “in-lipid mixing” method, and surface-engineered LNPs are prepared through different reactions. **E** HA-LNPs are prepared through NHS ester-amino reaction. **F** ASSET molecules are first inserted into LNPs, and surface-engineered LNPs are prepared following the addition of IgG antibodies. Created with BioRender.com

Since the post-insertion method requires the formation of micelles containing pre-synthesized ligands, macromolecule-based ligands such as antibodies that are not well tolerated to organic solvents used in the micelle preparation process are preferentially *in situ* conjugated to the preformed LNPs (Fig. 3). Depending on conjugation chemistry, *in situ* conjugation strategies can be divided into six classes: (1) thiol-maleimide (Mal) Michael reaction-based conjugation; (2) cyclopropene-Mal Diels-Alder reaction-based conjugation; (3) dibenzocyclooctyne (DBCO)-azide copper-free click reaction-based conjugation; (4) *N*-hydroxysuccinimide (NHS) ester-amine reaction-based conjugation; (5) affinity-based conjugation; and (6) reaction/affinity combination method.

Among these strategies, the thiol-Mal reaction is the most widely used method to conjugate ligands, especially antibodies, to the surface of LNPs (Fig. 3A–D). A general preparation procedure is briefly described as follows: Mal-functionalized LNPs are first prepared using the standard dilution method by incorporating Mal-PEG-DSPE in lipid mixtures or by post-inserting Mal-PEG-DSPE from micelles into LNPs. Then, antibodies are partially reduced by reducing agents such as tris (2-carboxyethyl)phosphine (TCEP) and dithiothreitol (DTT) to expose free reactive thiol groups for the conjugation step. For antibodies whose thiols are unavailable or absent, a protein modification agent, *N*-succinimidyl S-acetylthioacetate (SATA), is normally utilized to introduce external thiol groups into protein molecules. It works by reacting with accessible primary amine residues to install protected thiol groups on antibodies, followed by deprotection *via* hydroxylamine. Using the SATA method, various types of antibody-modified LNPs, including anti-CD4 (Tombácz *et al.* 2021), anti-CD5 (Rurik *et al.* 2022), anti-CD31 (Parhiz *et al.* 2018), anti-VCAM-1 (Vascular cell adhesion molecule 1) (He *et al.* 2022), and anti-CD117 (Breda *et al.* 2023), have been successfully developed and tested *in vivo*, demonstrating the feasibility and versatility of this method. In addition to thiol-Mal chemistry, Mal-PEG-DSPE was also used to fabricate targeted LNPs *via* the cyclopropene-Mal Diels-Alder reaction (Fig. 3D). For instance, a monovalent αPV1 (Fab-C4) containing a cyclopropene lysine (CpHK) at position 118 was conjugated to Mal-PEG-DSPE in a rapid and site-specific manner for producing lung-targeted LNPs, and the formed conjugates were found to exhibit the improved stability than the conventional thiol-Mal products (Li *et al.* 2020). However, Mal-PEG-DSPE is not always applicable, especially when LNPs are composed of disulfide bond-containing ionizable lipids, since the Mal groups might also be attacked by the thiol groups generated by the partly reduced lipids. Due to its fast kinetics and good stability in aqueous solutions, a DBCO-azide-based copper-free click reaction strategy, also termed the strain-promoted alkyne-azide cycloaddition (SPAAC), was utilized to attach ligands to the LNP surface in this case (Fig. 3D). To illustrate, a recent study has shown that azidated anti-podoplanin antibodies could be efficiently coupled to the DBCO-PEG-DSPE-bearing LNPs to achieve lymphatic endothelial cell targeting (Sakurai *et al.* 2022). For those targeting moieties that contain carboxylic or amine groups, the classic NHS ester-amine conjugation strategy can be applied for the surface modification of LNPs (Fig. 3D–E). For example, hyaluronic acids that have rich carboxylic groups were activated by the 1-ethyl-3-(3-dimethylaminopropyl) carbodiimide (EDC)/sulfo-NHS method and then coupled to amine-functionalized LNPs to achieve specific targeting of cluster of differentiation 44 (CD44)-expressing cells (Cohen *et al.* 2015; Singh *et al.* 2021). In another study, an oligopeptide MH42 that was found to facilitate the binding of the neural retina by *in vivo* bacteriophage biopanning was C-terminal lysine modified and then attached to LNPs bearing NHS ester-PEG-DSPE for subretinal delivery in a rhesus macaque non-human primate (NHP) model (Herrera-Barrera *et al.* 2023).

Despite great achievements, the aforementioned strategies are all based on covalent conjugation, which has some limitations, including high batch-to-batch variability, potential affinity reduction of antibodies, and the requirement for individual optimization for each antibody (Kedmi *et al.* 2018). To address these problems, Dan Peer’s group developed a flexible affinity-based antibody modification platform for LNPs termed ASSET (anchored secondary scFv enabling targeting) (Kedmi *et al.* 2018) (Fig. 3F). ASSET is a lipidated fusion protein consisting of an N-terminal signal sequence and an scFv (single chain fragment variable) of a monoclonal antibody (clone RG7/1.30) that specifically binds to the Fc region of rat IgG2a (RIg) antibodies. After expression and purification from bacteria, ASSET was first mixed with cholesterol to create micelles and then post-inserted into the preformed LNPs. Subsequently, RIg antibodies were added using an ASSET:RIg ratio of approximately 1∶1, and the RIg-decorated LNPs were formed in 30 min. Compared with chemical conjugation methods, ASSET exhibited an effective modification efficiency (~98%), maintained a high binding affinity (~0.24 nmol/L), and enabled the generation of distinct antibody-modified (such as anti-CD44, anti-CD34, anti-Ly6C, anti-CD3, anti-CD4, anti-CD25, anti-CD29, and anti-Itgb7) LNPs by simply replacing the type of RIg antibodies. In some specific applications, a combination of the above-mentioned methods might be needed. For example, for producing conformation-sensitive α4β7 integrin-targeted LNPs, RG7 antibodies were first conjugated to LNPs *via* thiol-Mal chemistry and then bound to the MAdCAM-1 (mucosal vascular addressin cell adhesion molecule-1)-Fc fusion proteins by affinity (Dammes *et al.* 2021). It was reported that the combination method was superior to the Thiol-Mal-based and the ASSET approach alone in this case, although the potential mechanism was not well understood.

## PURIFICATION AND CHARACTERIZATION TECHNIQUES

Pre-synthesized ligands that are incorporated by the “in-lipid mixing” method normally do not require a further purification step because it is expected that all the ligands interact with other lipid components for the self-assembly of LNPs. Regarding the ligands conjugated *in situ,* it is very crucial to remove the unbound molecules from the systems for obtaining high-purity surface-decorated LNPs, since the conjugation efficiency is relatively low and an excess amount of ligands is usually fed during the modification process. On one hand, free ligands might interfere with the subsequent characterization process and decrease the targeting efficiency by competitively binding to the relevant receptors *in vitro* and *in vivo*; on the other hand, adequate purification can promote the reproducibility of engineered LNPs, accelerating the bench-to-bedside translation and scale-up process. After purification, the final step before administration is the characterization of the engineered LNPs, which includes qualification/quantification of the attached ligands and validation of their functionalities. In order to understand the reality of functionalized LNPs in detail, a combination of diverse techniques is highly demanded. In this section, the current techniques that have been utilized to purify and characterize the surface-engineered LNPs are summarized (supplementary Table S1).

### Purification techniques

#### Dialysis

Dialysis is a diffusion-based separation technique that facilitates the transportation of molecules from higher to lower concentrations through a semi-permeable membrane (Schneiderman and Stalcup 2000). The semi-permeable membrane is placed between the sample and the dialysate, and it contains various-sized pores that allow molecules smaller than the pore size (also described as molecular weight cut-off, MWCO) to pass through. Dialysis is frequently applied to eliminate small molecular weight substances (*e*.*g*., SATA, TCEP, and DTT) and short peptides with membranes having small MWCO (500 Da–20 kDa). The membranes with high MWCO (such as 1 MDa) were also reported to remove macromolecules from LNPs, including HA (Cohen *et al.* 2015; Singh *et al.* 2021), lipidated peptide major histocompatibility complex type 1 (pMHC I) (Su *et al.* 2022), and lipidated ASSET fusion protein (Kedmi *et al.* 2018).

#### Ultracentrifugation

Ultracentrifugation is a combination of the application of centrifugal forces and filters containing semi-permeable membranes for the filtration of unwanted compounds based on their molecular weight (Nothnagel and Wacker 2018). Unlike dialysis, which requires the dialysate, ultracentrifugation works in the centrifuge tubes containing the filters and allows a rapid and convenient separation process. In addition, the whole purification process is performed in the same system, which minimizes the potential for contamination. Ultracentrifugation can be used to remove peptide-lipid conjugates and antibodies, and separate free siRNA from nanoparticles (He *et al.* 2022; Sakurai *et al.* 2016; Zhang *et al.* 2022); thus, it is widely utilized for the purification of siRNA-LNPs. However, the impact of centrifugal forces on the structure and integrity of LNPs during ultracentrifugation cannot be ignored and needs further investigation. Besides, a portion of LNPs can adhere to the filter membranes, causing sample loss and a low recovery rate.

#### Size exclusion chromatography

Size exclusion chromatography (SEC), also known as gel filtration chromatography, is the mildest chromatography technique that permits the effective separation of molecules by the difference of their molecular size in solution (Fekete *et al.* 2014). The column used in SEC is constructed of a porous matrix with a defined fractionation range, which determines the range of molecular weights (Mr) of the molecules that are accessible to the pores (Shanmuga Doss *et al.* 2017). After samples enter the porous matrix, larger molecules or particles are unable to penetrate the pores and thus elute first in the void volume (refers to the volume of the liquid phase between the matrix). Smaller molecules whose molecular weights are within the fractionation range are able to diffuse into the pores to varying extents depending on their size, with the smallest one diffusing deepest into the pores and therefore eluting last. By selecting appropriate pore sizes, SEC can be used for the removal of medium-to-large ligands, including peptides, aptamers, and antibodies from LNPs. For instance, a Sepharose CL-4B column that has a fractionation range between 60 and 20000 kDa was widely utilized to purify a series of IgG antibodies (~160 kDa) modified LNPs (Kedmi *et al.* 2018). Although the columns used in SEC are normally stable and non-reactive to most molecules, the potential interaction between the nanoparticles and porous matrix might still occur, which has to be carefully investigated.

### Characterization techniques

Once the purification is done, the final step is to characterize the incorporated ligands and validate their functions prior to administration. The characterization is normally performed in two aspects: the physicochemical characterization (*e.g.*, size, surface charge, and morphology) and the functional characterization. While general characterization methods are extensively used for measuring the physicochemical characteristics of modified LNPs, specific methods and techniques are required for testing the function of individual ligand types.

#### Physicochemical characterization techniques

Dynamic laser scattering (DLS) is one of the most widely used techniques to measure the hydrodynamic size, polydispersity, and surface charge of nanoparticles in suspension (Pecora 2000). The basic principle is relatively simple: the Brownian motioned nanoparticles are illuminated by a laser beam, and the fluctuations of the scattered light are detected and analyzed at a defined scattering angle (usually 173°), providing the diffusion coefficient that can be related to the hydrodynamic radius of a hypothetical hard sphere diffusing at the same rate as the tested particle through the Stokes-Einstein equation (Bhattacharjee 2016). The polydispersity index (PDI) that reflects the “non-uniformity” is also acquired spontaneously in the cumulant analysis of the DLS-measured intensity autocorrelation function (Farkas and Kramar 2021). With electrophoretic light scattering, the surface charge of nanoparticles can be further estimated by the measured electric potential around the particle at the slipping plane (Bhattacharjee 2016). With the DLS technique, a slight increase in hydrodynamic size was frequently observed in antibody-modified LNPs compared with unmodified ones, which indirectly confirmed the successful attachment of antibodies (Breda *et al.* 2023; Kedmi *et al.* 2018; Rurik *et al.* 2022). In addition, dropped zeta potentials were detected by the DLS measurement when polyanions such as aptamers and HA were conjugated to the LNP surface (Cohen *et al.* 2015; Liang *et al.* 2015; Singh *et al.* 2021). Similarly, significant positive and negative zeta potentials of SORT LNPs were detected when high ratios of permanently cationic lipids (*e.g.*, 1,2-dioleoyl-3-trimethylammonium propane, DOTAP) and anionic lipids (*e.g.*, 1,2-dioleoyl-sn-glycero-3-phosphate, 18PA) were incorporated, respectively (Cheng *et al.* 2020). However, DLS also has some drawbacks, including overestimation of larger particles and lack of information on particle concentration (James and Driskell 2013). The nanoparticle tracking analysis (NTA) technique that measures individual particle movement under a microscope is therefore used as a complementary method, which not only provides high-resolution sizing but also concentrations of measured samples (Hole *et al.* 2013). Besides, it can detect samples 10–1000 times more diluted than DLS, which is particularly suitable for detecting the fractions of low-concentration LNPs collected from the SEC purification step.

Small-angle X-ray scattering (SAXS) is another scattering-based analytical technique that measures the intensities of X-rays scattered by a sample as a function of the scattering angle (Li *et al.* 2016). SAXS measurements are typically performed at very small angles of 0.1–5^°^ and can provide structural information over a large range of length scales, from a couple of ångströms to hundreds of nanometers (Lombardo *et al.* 2020). This feature allows it to probe morphological details, from overall particle size to the lamellarity and internal structures of LNPs (Hammel *et al.* 2023). In addition, SAXS can be conducted in solution (Putnam *et al.* 2007), which allows for convenient evaluation of the influence of buffer compositions, environmental pH, and lipid components on the structure of LNPs. With the SAXS technique, the morphology of pSarcosinylated and PEGylated LNPs was investigated and compared (Nogueira *et al.* 2020). It was found that pSarcosinylation resulted in the formation of LNPs with a larger size and rougher surface but had little impact on the internal structure.

In addition to SAXS, transmission electron microscope (TEM) as a microscopy technique is also widely employed to visualize the morphology of LNPs (Arnould *et al.* 2018). For instance, a clear difference in the surface of LNPs before and after HA modifications was observed by TEM (Singh *et al.* 2021), while a maintained spherical shape of ligand-modified LNPs was imaged in the majority of cases. In another study, the integrity of glycosylated LNPs was investigated by negative staining TEM, where the incorporation of 10% and 15% of mannose-cholesterol was found to generate unstructured and irregularly shaped particles (Goswami *et al.* 2019). It is noteworthy that negative staining TEM can result in significant disruption of the morphology of lipid-based nanoparticles and cannot provide detailed information about their internal structures and contents. To address this issue, cryogenic transmission electron microscopy (cryo-TEM) was used to explore the detailed features of LNPs (Kulkarni *et al.* 2018). By instantly vitrifying samples, LNPs are preserved maximally in their near-native hydrated state (Friedrich *et al.* 2010). Using cryo-TEM, the unilamellar structure of scFv-functionalized LNPs with a diameter of 90 – 130 nm was clearly observed (Katakowski *et al.* 2016).

While the above-mentioned techniques are generally used to evaluate the overall structures and surface properties of modified LNPs, the techniques for the direct confirmation of attached ligands are demanded. Gel electrophoresis is a daily-used technique that can separate macromolecules such as DNA, RNA, and proteins by differences in their size and charge (Timms and Cramer 2008; Williams 2001), and it can be utilized to detect the conjugation of macromolecule-based ligands since the anchored ligands have increased size and migrate slower than the original ones in a gel. For example, polyacrylamide gel electrophoresis (PAGE) was utilized to analyze a CH6 aptamer before and after conjugation, and it revealed that the free CH6 aptamer moved faster than the coupled CH6-PEG-DSPE and CH6-siRNA-LNPs (Liang *et al.* 2015). In another case of spherical nucleic acid (SNA)-modified LNPs, the presence of bands at higher molecular weight than naked T21 DNA was observed in an agarose gel, which confirmed the surface DNA conjugation (Sinegra *et al.* 2021). Regarding antibody-modified LNPs, sodium dodecyl sulfate (SDS)-PAGE was commonly employed to validate the conjugation (Katakowski *et al.* 2016; Sakurai *et al.* 2022). SDS is an anionic surfactant that can disrupt the non-covalent bonds in proteins, allowing for proteins to be separated by their size (or molar mass) in a PAGE gel (Otzen 2011). Sakurai *et al.* have shown that the band with an increased molecular weight was clearly observed when the anti-podoplanin antibody was bound to LNPs *via* the DBCO-azide click reaction by SDS-PAGE (Sakurai *et al.* 2022). Likewise, a 3-kDa difference in migration of the scFv was resolved on PAGE gels, indicating the binding of the scFv to Mal-PEG-DSPE (Katakowski *et al.* 2016).

Since most ligands are based on peptides and proteins, colorimetric protein detection and quantification assays, such as the Bradford assay (Kruger 2009), bicinchoninic acid (BCA) assay (Walker 2009), and Peterson-Lowry assay (Peterson 1977), can be carried out to verify the conjugated products. In the specific case of modified LNPs, the BCA assay and Peterson-Lowry assay are preferred because the Bradford assay is not compatible with most surfactants, such as phospholipids, that are involved in every LNP formulation. The BCA assay is a high-throughput and sensitive colorimetric assay. It combines the reduction of the cupric cation (Cu^2+ ^) to the cuprous cation (Cu^+ ^) by proteins (particularly cysteines, cystines, tryptophans, tyrosines, and peptide bonds in proteins) in alkaline environments and the colorimetric detection of the purple-blue complexes formed by Cu^+ ^ and BCA (Walker 2009). According to the principle, reducing agents (*e.g.*, DTT and TCEP) that are frequently used in the antibody modification step can inflate protein concentration values by increasing the reduction of Cu^2+ ^, therefore requiring complete removal prior to the assay. In combination with the NTA technique, the BCA assay can even be employed to estimate the number of antibodies per LNP (Li *et al.* 2020). For instance, an average of 110 and 400–600 Fab-C4 antibodies were determined to exist for LNPs that were 70 and 160 nm in size, respectively (Li *et al.* 2020). The Peterson-Lowry assay, which works in a similar fashion to the BCA assay, was also reported to determine the concentration of conjugated anti-VCAM-1 antibodies (He *et al.* 2022). In this assay, the Folin-Ciocalteu reagent was added to the formed tetradentate-Cu^+ ^ complex, resulting in the formation of blue complexes that can be colorimetrically quantified (Peterson 1977). In addition to colorimetric assays, the conjugated ligands can also be detected or quantified by fluorescence and chemiluminescence methods. For example, a red fluorescent mCherry protein and a His-tag were fused to the C-terminal of ASSET, which enabled the validation of incorporated ASSET molecules by measuring mCherry fluorescence and horseradish peroxidase (HRP) substrate chemiluminescence following the sequential labeling with anti-His-tag antibody and HRP-conjugated secondary antibody (Kedmi *et al.* 2018). Notably, the HRP-conjugated antibodies were also used in western blot and dot-blot analyses for detecting antibody-functionalized LNPs (Kedmi *et al.* 2018). To quantify the number of MH42 peptides on LNPs, a fluorometric maleimide assay was performed to detect free maleimide groups before and after the thiol-maleimide reaction (Su *et al.* 2022). It was calculated that 376 and 492 pg of MH2 peptide existed per microliter of LNPs, where 0.15% and 0.3% of PEG-lipids were substituted with Mal-PEG-DSPE, respectively. Regarding the quantification of other types of ligands, some specific methods might be needed. To illustrate, tritium (^3^H)-labeled HA was used to quantify the conjugated HA on LNPs, and it was reported that the concentration of 75 μg HA/μmole lipid was typically achieved (Cohen *et al.* 2015).

#### Functional characterization techniques

Once the presence of ligands on the LNP surface has been confirmed, it is necessary to validate their functionalities, in most cases, their targeting ability, prior to the *in vivo* studies. Functional characterization techniques are mainly divided into two primary classifications: cell-free assays and *in vitro*/*ex vivo* cell-based assays. Specific methods for testing certain ligand types and general techniques that are frequently utilized are summarized below.

The lectin affinity assay is a classical and widely used cell-free technique for testing the functional binding of glycosylated lipids (Smith and Virginia Torres 1989). The glycolipids are first bound to different immobilized lectins that have affinity for specific sugar moieties and then eluted by the sugar-containing buffer for further quantitative analysis. In a recent study, Kasiewicz *et al.* used this technique to confirm the incorporation of a synthetic GalNAc-PEG-lipid and showed that an additional peak appeared in the HPLC (high-performance liquid chromatography) chromatogram with the eluted LNP fractions from the lectin affinity column (Kasiewicz *et al.* 2023). In addition to the galactose, the lectin concanavalin A (ConA) binding assays were also employed to measure the presentation of mannose-cholesterol ligands on LNPs, where M3 ligands that contain three repeated mannose units exhibited the highest recognition (Goswami *et al.* 2021). Instead of using lectins, Akinc *et al.* utilized a soluble dendritic cell ASGPR (asialoglycoprotein receptor) and a fluorescein-labeled triantennary GalNAc reporter probe to establish a cell-free receptor competition method for determining the binding activity of GalNAc-LNPs (Akinc *et al.* 2010), and it was found that the binding pattern was GalNAc-dependent, with no appreciable binding detected for unmodified LNPs. To visualize the binding specificity and kinetics of alendronate-conjugated bone-targeted LNPs (BP-LNPs), a lipophilic carbocyanine dye DiO was incorporated into LNPs, and the binding assays were performed with hydroxyapatite (HA), which is the main component of bone (Xue *et al.* 2022). In contrast to unmodified LNPs, a faster and more efficient binding of BP-LNPs to HA was observed, with 80% HA binding efficiency achieved after a 2-hour incubation. The binding activity of antibody-based ligands is commonly measured by the enzyme-linked immunosorbent assay (ELISA), which uses solid-phase immobilized antigens to detect the antibodies of interest in a liquid sample (Malvano *et al.* 1982). For instance, the functional binding of anti-PV1-modified LNPs towards its antigen PV1 was measured by ELISA, with EC_50_ values of 3.6 ± 0.4 and 4.5 ± 0.4 ng/mL detected for 70 and 160 nm anti-PV1-LNPs, respectively (Li *et al.* 2020). In another example, the binding affinity of the incorporated ASSET and the bound anti-CD34 antibodies were assayed, and it was found that anti-CD34 antibodies conjugated by the ASSET platform could maintain their high affinity with an average K_d_ (equilibrium dissociation constant) of ~0.24 nmol/L detected by ELISA (Kedmi *et al.* 2018).

While affinity-based assays are only able to confirm the maintenance of ligand binding activity after conjugation, techniques that could validate the ability to mediate receptor-specific cellular binding and uptake are required. Flow cytometry and confocal laser scanning microscopy (CLSM) are two broadly used techniques for this purpose. Flow cytometry allows a combined assessment of cell morphology and physiology *via* scatter light and a fluorescent readout by using fluorescent dye-labeled nucleic acids and/or fluorescent molecules (*e.g.*, DiI, DiD, and DiO) (McKinnon 2018). CLSM uses basically the same fluorescent components as flow cytometry, but it produces high-resolution and high-contrast images for directly visualizing the cells by means of using a spatial pinhole to block out-of-focus light (Wang *et al.* 2012). Flow cytometry provides multi-parametric analysis of individual cells in cell suspension, while CLSM enables visualization of nanoparticle distribution at the cellular and subcellular levels in culture environments (Ducat *et al.* 2011). In the case of accurate quantification, flow cytometry outperforms CLSM, but normally they are both carried out to provide complementary information. For example, Sakurai *et al.* developed an Epi-1 peptide-modified multifunctional envelope type nanodevice called Pep-MEND, which targets the epithelial cell adhesion molecule (EpCAM) (Sakurai *et al.* 2020). The cellular uptake of lipid envelope and siRNA by the dye-labeled Pep-MEND was significantly higher than the unmodified PEG-MEND in EpCAM-expressing SK-OV-3 cells by flow cytometry. To further confirm that the Pep-MEND was internalized and not bound to the cell membranes, a CLSM study was performed. Compared with the PEG-MEND, a larger amount of Cy5-siRNA was observed inside cells treated with the Pep-MEND, with most of them not colocalized with endosomes, which indicated efficient endosomal escape of the Pep-MEND. In addition to the intracellular trafficking of LNPs, CLSM was also utilized to explore the endocytic pathways of CH6 aptamer-modified osteoblast-targeted LNPs (Liang *et al.* 2015). Different LNP formulations containing Cy3-siRNA were applied to osteoblasts, and various endocytic markers were labeled with Alexa-Fluor 488 for the co-localization analysis. The results showed that most of the siRNA in CH6-LNPs co-localized with transferrin (a marker for clathrin-mediated endocytosis) and dextran (a marker for macropinocytosis), but not with cholera toxin (a marker for caveolae-mediated endocytosis) in osteoblasts, suggesting that clathrin-mediated endocytosis and macropinocytosis played a predominant role in the internalization of CH6-LNPs. In contrast, unmodified and random sequence-modified LNPs were found to be taken up only *via* the clathrin-mediated endocytosis. Besides *in vitro* cell-based assays, an *ex vivo* bone model was also established for the affinity evaluation of BP-LNPs, where the bone fragments were first treated with free DiO, unmodified DiO-LNPs, and DiO-BP-LNPs, and then the fluorescence intensities were detected (Xue *et al.* 2022). Under fluorescence microscopy, minimal to weak fluorescence signals were observed on the bone samples incubated with free DiO and DiO-LNPs. On the contrary, the BP-LNPs-treated bone fragments exhibited a strong DiO signal, which indicated that the incorporation of alendronate-lipids could facilitate nucleic acid delivery to the bone.

## PURPOSES OF SURFACE ENGINEERING

Following systemic administration, LNPs undergo desorption of PEG-lipids and adsorption of soluble ApoE on their surface, which facilitates hepatic entry by the improved binding to LDLR that is heavily expressed on the sinusoidal surface of hepatocytes (Akinc *et al.* 2010). To expand the scope of LNPs to non-hepatic cells, Cheng *et al.* developed selective organ targeting (SORT) nanoparticles and achieved spleen- and lung-selective mRNA delivery by incorporating anionic phospholipids and permanently cationic lipids into conventional four-component LNPs, respectively (Cheng *et al.* 2020). SORT LNPs were reported to follow a similar mechanism to conventional LNPs for organ targeting, but they adsorbed different types of serum proteins by distinct surface charges, which might direct them to non-liver organs (Dilliard *et al.* 2021). Different from endogenous targeting *via* serum proteins, numerous ligands were utilized to functionalize LNPs to achieve targeted nucleic acid delivery through active targeting*.* In this section, we summarize the currently reachable organs and cell types by ligand-modified nucleic acid-LNPs (supplementary Table S2).

### Targeted nucleic acid delivery

#### Targeting liver cells

**Figure 4:**
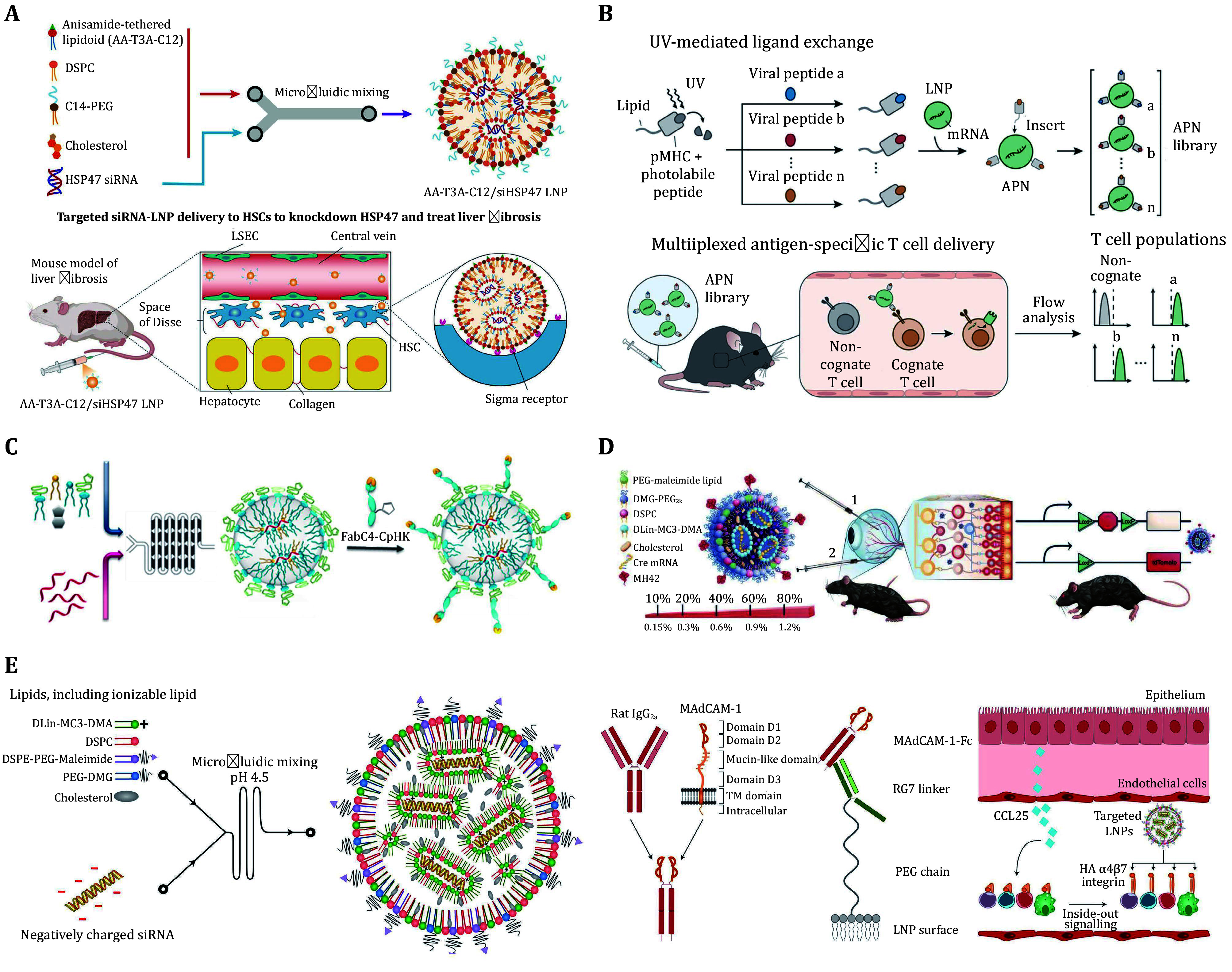
Surface-engineered LNPs for targeted nucleic acid delivery. **A** Formulation of AA-T3A-C12/siHSP47 LNP *via* microfluidic mixing and schematic illustration of targeted delivery to activated HSCs to knockdown HSP47 and treat liver fibrosis. Adapted from Han *et al.* (2023) with permission. **B** Schematic illustration of UV-mediated peptide exchange of MHCI APNs for *in vivo* multiplexed delivery to virus-specific T cells. Adapted from Su *et al.* (2022) with permission. **C** Schematic illustration of αPV1-LNPs construction *via* microfluidic mixing. Adapted from Li *et al.* (2020) with permission. **D** Schematic illustration of LNP formulation and conjugation with peptide *via* maleimide-thiol chemistry and Cre mouse model depicting both routes of administration trialed. Adapted from Herrera-Barrera *et al.* (2023) with permission. **E** Generation of LNPs to target the high-affinity conformation of integrin α4β7. Adapted from Dammes *et al.* (2021) with permission

As discussed above, nucleic acid-LNPs have demonstrated utilities in the treatment of liver diseases since they are mainly taken up by hepatocytes in the liver *via* LDLR-mediated endocytosis (Akinc *et al.* 2010). However, for patients with homozygous familial hypercholesterolemia, the LDLR activity is low and may not be sufficient to mediate the efficient hepatic entry of administered LNPs (Kasiewicz *et al.* 2023). Therefore, an alternative to endogenous ApoE-based targeting is highly desired. The first exogenous targeting strategy for the liver was explored for siRNA delivery using a trivalent GalNAc-PEG-lipid that targets the ASGPR (Akinc *et al.* 2010). It was found that GalNAc modification was able to rescue the potency of LNPs in the ApoE^−/−^ mice in a GalNAc-dependent manner. Furthermore, GalNAc-LNPs exhibited comparable activity in wild-type and LDLR^−/−^ mice, indicating that the GalNAc-PEG-lipid can serve as an alternative and effective ligand to allow efficient internalization of LNPs by hepatocytes. More recently, this strategy was further validated in an LDLR-deficient non-human primate (NHP) model, where LNPs modified with a rationally designed ASGPR-targeted GL6 ligand effectively delivered adenine base editing mRNA to the liver, leading to efficient base editing of the ANGPTL3 (angiopoietin-like 3) gene (Kasiewicz *et al.* 2023). While both ApoE-based endogenous targeting and GalNAc-based exogenous targeting enable potent LNP delivery to hepatocytes, targeted and efficient delivery to activated hepatic stellate cells (HSCs) remains a challenge. Inspired by a small molecule anisamide that targets the sigma receptor overexpressed in activated HSCs, Han *et al.* synthesized a series of anisamide-tethered lipidoids (AA-lipidoids) and identified a top performer, AA-T3A-C12, with both high potency and selectivity for activated HSC transfection (Han *et al.* 2023) (Fig. 4A). In a liver fibrosis mouse model, AA-T3A-C12 LNPs carrying HSP47 (heat shock protein 47) siRNA achieved ~65% gene silencing in activated HSCs, resulting in significantly reduced collagen deposition and liver fibrosis. Apart from anisamide, a peptide-based targeting moiety called pPB was also incorporated into LNPs for activated HSC targeting (Jia *et al.* 2018). The pPB peptide is a cyclic oligopeptide that has a strong binding affinity to the platelet-derived growth factor receptor β that is overexpressed in activated HSCs. The results showed that pPB-modified LNPs exhibited increased HSC uptake and enhanced gp46 (the rat homologue of human HSP47) silencing *in vivo*. Using the same ligand, Zhang *et al.* constructed pPB-siRNA-LNPs targeting the high mobility group box-1 (HMGB1) protein that can promote the development of both hepatic inflammation and fibrosis. Compared with HSP47 siRNA that has only an antifibrotic effect, the pPB-HMGB1 siRNA-LNPs that possess dual antifibrotic and anti-inflammatory effects efficiently inhibited HSC proliferation, collagen deposition, and fibrosis formation in the liver, significantly prolonging the survival time of cirrhotic mouse models (Zhang *et al.* 2020). In addition to hepatocytes and HSCs, mannose-modified LNPs (M-LNPs) were fabricated to target liver macrophages that highly express the mannose receptor (Wang *et al.* 2023b), and the M-LNPs loaded with tumor necrosis factor α (TNFα) siRNA remarkably suppressed TNFα expression and improved liver damage in an acute liver injury mouse model.

#### Targeting spleen and immune cells

The spleen is the largest secondary lymphoid organ and plays a major role in filtering blood-borne pathogens and antigens as well as regulating immune responses (Lewis *et al.* 2019). Since the spleen is a rich resource of large numbers of immune cells (Bronte and Pittet 2013), developing spleen- or specific splenic cell type-targeted LNPs holds great potential for preventing and treating immune-related diseases, such as cancers, infectious diseases, autoimmune diseases, and pathogenic inflammatory disorders (Wang *et al.* 2023a). For example, LNPs decorated with a single-chain antibody that is specific for the dendritic cell (DC) receptor DEC205 were developed for targeting DCs *in vivo* (Katakowski *et al.* 2016). Intravenous injection of DEC205-LNPs containing CD45, CD80, and CD86 siRNA resulted in the preferential internalization of LNPs in splenic DCs, leading to the reduced expression of these costimulatory molecules. In addition to DCs, LNPs have also been extensively engineered to target T lymphocytes in the spleen. Tombácz *et al.* reported anti-CD4 antibody-modified LNPs that enabled specific mRNA delivery to CD4+ T cells (Tombácz *et al.* 2021). Upon systemic injection, radiolabeled anti-CD4-LNPs mainly accumulated in the spleen, achieving ~30-fold higher signal of reporter mRNA in CD4+ T cells than unmodified LNPs. Moreover, anti-CD4-LNPs containing Cre recombinase-encoded mRNA enabled genetic recombination in about 60% of CD4+ T cells in the spleen, with no variability in the uptake of LNPs in different CD4+ T cell subpopulations. To avoid the high cost and complex manufacturing of *ex vivo*-based chimeric antigen receptor (CAR) T-cell therapy, anti-CD5-modified LNPs were developed to produce CAR T cells *in vivo* (Rurik *et al.* 2022). It was reported that anti-CD5-LNPs encapsulating the CAR for fibroblast activation protein (FAP) (a marker of activated fibroblasts) were able to induce FAPCAR expression in each major T cell subset with 87% and 9%–10% of CD4+ and CD8+, respectively. In a mouse model of heart failure, anti-CD5-LNPs successfully generated antifibrotic CAR T cells *in vivo*, resulting in reduced fibrosis and restored cardiac function. Besides anti-CD5 antibodies, anti-CD3 antibodies were also decorated on LNPs for the production of CAR T cells *in vivo* (Zhou *et al.* 2022). To illustrate, plasmid DNA that encodes both interleukin 6 short hairpin RNA (*IL-6* shRNA) and CD19-CAR was loaded into anti-CD3-LNPs, and the LNPs enabled the generation of CAR T cells with *IL-6* gene knockdown *in vivo*, which not only killed the CD19-highly expressed leukemia tumor cells but also alleviated cytokine release syndrome (CRS) caused by *IL-6*. Due to the diversity of peptide epitopes and polymorphisms of class I major histocompatibility complexes (MHCI), multiplexed mRNA delivery to various populations of antigen-specific CD8+ T cells remains a significant challenge. To achieve it, Su *et al.* developed light-induced peptide MHCI (pMHCI)-exchangeable antigen-presenting LNPs (APNs) by incorporating lipidated photoswitchable pMHCI molecules (Su *et al.* 2022) (Fig. 4B). *In vivo* studies showed that distinct APNs targeting three immunodominant epitopes were able to achieve ~50% transfection of their cognate antigen-specific CD8+ T cells in a PR8 influenza model. In addition to antibodies and pMHCI molecules, G-rich DNA was also reported to promote the uptake of LNPs to the spleen *via* a class A scavenger receptor-mediated endocytosis mechanism (Sinegra *et al.* 2021).

#### Targeting lung cells

Nucleic acid drugs offer gene therapy for the treatment of various lung diseases such as asthma, pulmonary fibrosis, acute lung injury, pulmonary hypertension, and lung cancer (Chen *et al.* 2018). However, the delivery of genetic materials to the lungs by conventional LNPs is inefficient, which might be due to low LNP uptake by pulmonary cells and remarkable airway defenses (Kubczak *et al.* 2021). Jin *et al.* developed three types of siRNA-LNPs (native, cationic, and mannose-incorporated LNPs) and evaluated their pulmonary delivery efficiency *via* intratracheal injection (Jin *et al.* 2023). It was found that mannosylated LNPs exhibited the most potent delivery of siRNA to the three different lung cell types (epithelial, endothelial, and immune cells) *in vivo*. In a pulmonary fibrosis mouse model, mannosylated LNPs encapsulating G2 and S phase expressed protein 1 (GTSE1) siRNA significantly decreased collagen accumulation and down-regulated the epithelial-mesenchymal transition (EMT) associated-protein in the lungs, leading to functional recovery from pulmonary fibrosis. To specifically target lung endothelial cells, Parhiz *et al*. developed anti-CD31 (also known as platelet endothelial cell adhesion molecule-1, PECAM-1) functionalized LNPs and found that intravenous injection of anti-CD31-LNPs resulted in 200-fold and 25-fold enhancement of mRNA delivery and protein expression in the lungs compared to nonfunctionalized LNPs, respectively (Parhiz *et al.* 2018). In addition to CD31, plasmalemma vesicle-associated protein 1 (PV1), a known caveolae-associated protein, was also utilized as a target for re-directing LNPs to the endothelial membranes in lung blood capillaries (Li *et al.* 2020). Similar to anti-CD31-LNPs, systemic administration of anti-PV1-LNPs markedly increased mRNA delivery to the lungs and achieved 40-fold higher protein expression than the isotype control (Li *et al.* 2020) (Fig. 4C). Given that CD54 (also known as intercellular adhesion molecule-1, ICAM-1) is upregulated by inflammatory stimuli on airway epithelial cells (AECs) in asthma (Inoue *et al.* 2020), a cyclic peptide (amino acid sequence: CSERSMNFC) ligand that efficiently binds to the CD54 receptor was chosen to construct AEC-specific siRNA-Pep-LNPs against thymic stromal lymphopoietin (TSLP) (Zhang *et al.* 2022). Upon pulmonary administration, Pep-LNPs efficiently delivered siRNA to the mouse AECs and significantly reduced the expression of TSLP, leading to remarkable alleviation of the inflammatory response and airway hyperresponsiveness (AHR) in asthmatic mice.

#### Targeting tumor cells

Passive targeting that is based on the so-called enhanced permeation and retention (EPR) effect is substantially utilized in cancer nanomedicines for improving the accumulation of therapeutic nanocarriers at the tumor site (Torchilin 2011). PEGylation is the most widely used strategy to achieve passive targeting (Fang *et al.* 2011), but a low-shedding PEG-lipid (such as PEGylated distearyl lipid) and relatively high-density modification are often required. For example, 5% of PEG-DSPE is incorporated for the preparation of the FDA-approved liposomal chemotherapeutic drug Doxil^®^ (Barenholz 2012). However, in the case of LNPs, the mentioned ratio and type of PEG-lipids were not able to generate effective formulations for *in vivo* delivery of nucleic acid drugs (Akinc *et al.* 2010; Chen *et al.* 2016), probably due to the low cellular uptake and insufficient endosomal escape. Therefore, an active targeting moiety that can enhance the permeation and internalization of LNPs into tumor cells is highly demanded (Nakamura *et al.* 2022). HA, the major ligand of the CD44 receptor overexpressed on various types of tumor cells, has been broadly used to construct CD44-targeted nanoparticles (Jordan *et al.* 2015), including nucleic acid-LNPs. For example, HA-decorated LNPs loaded with polo-like kinase 1 (PLK1) siRNA significantly reduced PLK1 mRNA levels by more than 80% in CD44 receptor-expressing glioblastoma multiforme (GBM) cells, which dramatically prolonged the survival time of treated mice in a human GBM U87MG orthotopic xenograft model (Cohen *et al.* 2015). In another advanced orthotopic ovarian cancer model, intraperitoneal administration of HA-LNPs loaded with a combination of eukaryotic translation-initiation factor 3c (eIF3c) and PLK1 siRNA resulted in robust gene silencing in tumor tissues, with a remarkably enhanced overall survival of 60% achieved compared with unmodified and single siRNA-loaded LNPs (Singh *et al.* 2021). CD38 is another surface marker that is overexpressed in several types of tumors, such as mantle cell lymphoma (MCL) and multiple myeloma (MM) (Morandi *et al.* 2018; Ye *et al.* 2017). Weinstein *et al.* have shown that anti-CD38 antibody-coated LNPs were able to be specifically taken up by human MCL cells in the bone marrow of xenografted mice (Weinstein *et al.* 2016). When encapsulating cyclin D1 siRNA, anti-CD38-LNPs efficiently induced gene silencing and suppressed tumor cell proliferation *in vivo*, prolonging the survival of MCL-bearing mice. The tumor-targeting ability of anti-CD38-LNPs was also validated in a xenograft MM mouse model, where specific delivery of cytoskeleton-associated protein 5 (CKAP5) siRNA to bone marrow-residing and disseminated MM cells and improved therapeutic outcomes were achieved (Tarab-Ravski *et al.* 2023). In addition to CD38, the epidermal growth factor receptor (EGFR) can also serve as a target for tumor cell-specific delivery of therapeutic nucleic acids. For instance, anti-EGFR-LNPs loaded with Cas9 mRNA and PLK1 sgRNA enabled ~82% of PLK1 gene editing in human serous ovarian adenocarcinomas Ovcar8 (OV8) cells, which strongly inhibited tumor growth and increased overall survival by ~80% in metastatic OV8-bearing mice (Rosenblum *et al.* 2020). When loaded with siRNA against HPV16-E6/E7, the superior tumor suppression effect of anti-EGFR-LNPs was also observed, with a 50% greater reduction of tumor volume that was achieved compared to isotype control LNPs in a xenograft HPV-positive tumor model (Kampel *et al.* 2021). By modifying the surface of the anti-PD-L1 antibody, LNPs could be further engineered to spontaneously target tumor cells and tumor myeloid cells (Yong *et al.* 2022). When loaded with siRNA against heme oxygenase-1 (*HO1*), a chemoresistance-related molecule in tumor cells and an immunotherapeutic molecule in tumor myeloid cells, anti-PD-L1-LNPs were able to induce *HO1*-inhibition in tumor cells to sensitize chemotherapeutics and reprogram tumor myeloid cells to drive the “cold-to-hot” transition, resulting in an improved response to anti-PD1 antibody therapy in a triple combination study.

Apart from antibodies, a variety of peptides were modified on LNPs to achieve specific tumor cell targeting, among which the cyclic RGD (cRGD) peptide that recognizes the αvβ3 integrin overexpressed in tumor endothelial cells (TECs) is the most studied (Asati *et al.* 2019). Sakurai *et al.* utilized cRGD-LNPs to remodel the extracellular matrix (ECMs) in the tumor microenvironment by delivering vascular endothelial growth factor receptor 2 (*VEGFR2*) siRNA (Sakurai *et al.* 2016). It was found that cRGD-LNPs induced the efficient inhibition of VEGFR2 on TECs and caused the infiltration of macrophages and the subsequent degradation of the ECMs, which allowed nanoparticles to penetrate more deeply into the tumor tissues. The cRGD-LNPs were also utilized to treat metastatic cancer by delivering delta-like ligand 4 (*DLL4*) siRNA (Sakurai *et al.* 2018). The systemic injection of cRGD-LNPs induced robust DLL4 gene silencing in the metastasized vasculature in the lungs, dramatically prolonging the overall survival of metastasized model mice. Apart from TECs, cRGD can be also utilized to directly target tumor cells that overexpress αvβ3 integrin, such as HepG2 liver cancer cells. It was reported that cRGD-modified iLNPs could interact with HepG2 cells more efficiently than unmodified counterparts, which dramatically enhanced antitumor efficacy of *PLK1* siRNA in tumor-bearing mice (Guo *et al.* 2021). The truncated Lyp-1 (tLyp-1) peptide is another tumor-targeting ligand that has also shown excellent vascular permeation and tumor-homing capacities (Timur and Gürsoy 2021). Anthiya *et al.* designed pan KRAS (Kirsten rat sarcoma viral oncogene homolog) siRNA-loaded tLyp-1 modified LNPs and have shown that tLyp-1-LNPs exhibited enhanced accumulation in the tumor tissues compared to their unmodified counterparts (Anthiya *et al.* 2023). Moreover, in combination with a classical chemotherapeutic agent gemcitabine, a remarked reduction in tumor growth was achieved in pancreatic cancer-bearing mice treated with tLyp-1-LNPs. The Epi-1 peptide, a nonstandard macrocyclic peptide discovered by a random nonstandard peptides integrated discovery (RaPID) system, has shown a high affinity to the epithelial cell adhesion molecule (EpCAM) that is expressed in several types of tumors and was thus used to construct EpCAM-targeting LNPs for the treatment of EpCAM-positive cancers. When systemically injected, Epi-1-LNPs carrying PLK1 siRNA achieved significant inhibition of PLK1 gene expression at tumor sites in a Hep3B xenograft cancer model (Sakurai *et al.* 2017) and an ovarian cancer peritoneal dissemination model (Sakurai *et al.* 2020). To reverse the immunosuppressive tumor microenvironment caused by the loss of the p53 gene in hepatocellular carcinoma (HCC), Xiao *et al.* developed a CTCE peptide-conjugated CXCR4 (a validated selective target in HCC)-targeted LNP for the efficient encapsulation and HCC-selective delivery of p53 mRNA (Xiao *et al.* 2022). In p53-null orthotopic and ectopic models of murine HCC, the CTCE-decorated nanocarriers combined with anti-PD-1 therapy enabled global reprogramming of the immune TME, leading to improved tumor inhibition effects compared to individual therapy alone.

In addition to the above-mentioned antibodies and peptides, small molecule- and aptamer-based targeting ligands were also reported to engineer LNPs for tumor targeting. For example, glutamate-urea-lysine (Glu-urea-Lys), which binds to the prostate-specific membrane antigen (PSMA) with high affinity, was conjugated to PEG-lipids for fabricating targeted LNPs in the treatment of prostate cancer (Lee *et al.* 2016). When formulated with siRNA against the androgen receptor, the Glu-Urea-Lys-LNPs induced enhanced accumulation and cellular internalization at tumor sites, leading to down-regulation of androgen receptor levels and inhibition of tumor cell proliferation in a mouse xenograft model. To develop an osteoblast-specific delivery system, an aptamer called CH6 was screened and identified as a highly potent osteoblast-targeting ligand by the cell-based systematic evolution of ligands by exponential enrichment (cell-SELEX) (Liang *et al.* 2015). The CH6 aptamer-functionalized LNPs encapsulating osteogenic pleckstrin homology domain-containing family member 1 (*Plekho1*) siRNA boosted osteoblast-specific gene silencing and facilitated bone formation in both osteopenic and healthy rodents.

#### Targeting other organs and cell types

The blood-brain barrier (BBB) prevents the entry of most macromolecule drugs from the blood into the brain and has been a great hurdle for brain delivery of therapeutic nucleic acids (Pardridge 2007). To address this issue, RVG (rabies virus glycoprotein)-9r peptide-modified LNPs were developed and loaded with siRNA against mutant ataxin-3, a Machado-Joseph disease (MJD) causing protein, for testing their therapeutic effect in a MJD mouse model (Conceição *et al.* 2016). Intravenously administered RVG-9r-LNPs induced efficient silencing of mutant ataxin-3 while preserving wild-type ataxin-3 in neuronal cells of the brain, resulting in the alleviation of neuropathology and motor behavior deficits *in vivo*. In the treatment of another brain disease, acute ischemic stroke (AIS), Jia *et al.* prepared IL-10 mRNA-loaded anti-VCAM-modified LNPs targeting the BBB endothelium and systemically injected them into the transient middle cerebral artery occlusion (tMCAO) mouse model (Jia *et al.* 2023). The results showed that anti-VCAM-LNPs achieved nearly two orders of magnitude higher brain delivery than their unmodified counterparts, which led to a 73% reduction in cerebral infarct volume and a lower mortality rate. It is worth noting that the incorporation of an additional cationic lipid, DOTAP, might further improve the endothelial association and transfection efficiency of anti-VCAM-LNPs (He *et al.* 2022). While LNPs were able to mediate mRNA delivery to the retinal pigment epithelium (RPE) and Müller glia, delivery to the photoreceptors (PRs) still remains a significant challenge. To overcome the ocular barriers and penetrate the neural retina, Herrera-Barrera *et al.* developed novel peptide-guided LNPs that were modified with the MH42 peptide discovered by *in vivo* bacteriophage peptide biopanning (Herrera-Barrera *et al.* 2023) (Fig. 4D). It was found that MH42-LNPs enabled mRNA delivery to the PRs, RPE, and Müller glia in both mice and non-human primates, providing a promising strategy for the treatment of inherited blindness. In addition to the MH42 peptide, a most recent study reported that LNPs containing carboxy-ester PEG-lipids (LNPx), compared to other PEG variants, displayed improved pan-retinal distribution in the photoreceptors and RPE upon subretinal administrations (Gautam *et al.* 2023). By incorporating Cas9 mRNA and sgAi9, 16.4% of editing efficiency was achieved in RPE *in vivo*. Similar to the brain and retina, the bone is also a hard-to-reach organ for LNPs due to several biological barriers (Lavrador *et al.* 2018), including relatively low bone blood flow, the bone marrow barrier, and the low binding affinity of LNPs to bone minerals (hydroxyapatite, HA). Inspired by alendronate, a bisphosphonate medication for bone disorders that has a high affinity for HA, Xue *et al.* synthesized a series of bisphosphonate (BP)-lipidoids that possess enhanced bone-binding ability and formulated them with other lipid excipients into LNPs (Xue *et al.* 2022). The identified lead formulation 490BP-C14 LNPs exhibited improved LNP accumulation and mRNA expression in the bone and enabled the secretion of therapeutic bone morphogenetic protein-2 from the bone microenvironment upon systemic injection.

In addition to the major organs, LNPs have been greatly engineered to target various specific cell types. As mentioned above, an ASSET platform was developed for redirecting LNPs to diverse leukocyte subsets *in vivo*, including CD44-, CD34-, Ly6C-, CD3-, CD4-, CD25-, CD29-, and Itgb7-positive cells, by simply switching different types of IgG antibodies (Kedmi *et al.* 2018). Besides this modular platform, the traditional Thiol-Mal reaction was frequently used to construct LNPs for leukocyte targeting. For example, a robust gene silencing effect in primary leukocytes was achieved by LNPs modified with a pan leukocyte selective targeting agent (β7 integrin) (Ramishetti *et al.* 2020). Furthermore, conformation-sensitive targeted LNPs were further fabricated by the combination of thiol-Mal-mediated RG7 conjugation and affinity-induced MAdCAM-1 binding (Dammes *et al.* 2021) (Fig. 4E). In a colitis mouse model, the formed LNPs were found to specifically target inflammatory gut-homing leukocytes *via* the high-affinity conformation of α4β7 integrin, leading to enhanced silencing of interferon-γ in the gut and improved therapeutic effects in colitis. When conjugated with anti-CD4 antibodies, the generated LNPs were able to efficiently bind and enter CD4+ T in several anatomical sites (such as the spleen, inguinal lymph nodes, blood, and the bone marrow), and a silencing effect on circulating and resting CD4+ T lymphocytes was observed *in vivo* (Ramishetti *et al.* 2015). Although anti-VCAM antibodies were able to redirect LNPs to vascular endothelial cells as discussed above, the specific targeting of lymphatic endothelial cells (LECs) that are from lymphatic vessels is still a challenge. To achieve specific siRNA delivery to LECs, Sakurai *et al.* developed targeted LNPs that were coupled with anti-podoplanin (a marker of LECs) antibodies *via* a click reaction-based approach called CLIP (Sakurai *et al.* 2022). The CLIP approach enabled fast and efficient preparation of anti-podoplanin-LNPs, and the intratumorally injected LNPs were taken up by LECs both in the tumor tissues and draining lymph nodes. Hematopoietic stem cells (HSCs) that reside in the bone marrow are the source of all cells in the blood and immune system through hematopoiesis (Ding and Morrison 2013). Hematopoietic disorders can be treated by replacing diseased HSCs with healthy or genome-edited HSCs through HSC transplantation (Hatzimichael and Tuthill 2010). However, the generation of these HSCs in the process of *ex vivo* gene therapy is complicated, and the transplantation of HSCs requires “conditioning” regimens that cause substantial acute and chronic systemic toxicities in patients (Casper *et al.* 2004). To solve these limitations, HSC-targeted LNPs modified with antibodies against CD117 (also known as c-Kit) were developed (Breda *et al.* 2023; Shi *et al.* 2023). Intravenous administration of anti-CD117-LNPs enabled efficient and specific delivery of multiple types of nucleic acids, including siRNA and mRNA, to HSCs *in vivo* (Breda *et al.* 2023; Shi *et al.* 2023). Moreover, when loaded with pro-apoptotic PUMA (p53 up-regulated modulator of apoptosis) mRNA, anti-CD117-LNPs induced significant HSC depletion, which allowed for the successful engraftment of BM cells (Breda *et al.* 2023). It has been discussed above that mannosylation is capable of enhancing the uptake of LNPs by liver macrophages and lung cells (Jin *et al.* 2023; Wang *et al.* 2023b). Gao *et al.* showed that mannosylated LNPs were also able to facilitate IL-10 mRNA delivery to atherosclerotic lesional M2-like macrophages and subsequently induce efficient expression and secretion of IL-10 (Gao *et al.* 2023). Furthermore, it was found that the secreted IL-10 inhibited the expression of pro-inflammatory cytokines, reduced necrotic areas, and increased fibrous cap thickness, which altogether led to significant therapeutic effects in a Western diet-fed LDLR^−/−^ mouse. Lyophilization and topical application of the surface-engineered LNPs were also investigated by Li *et al.*, where keratinocyte-targeted peptide A5G33-modified LNPs encapsulating locked nucleic acid (LNA)-modified anti-miR-107 were fabricated, lyophilized, and dispersed in hydrogel for topical application in mice bearing burn wounds (Li *et al.* 2018). The results showed that lyophilized keratinocyte-targeted LNPs enabled the depletion of miR-107 and promoted differentiation of keratinocytes, resulting in acceleration of wound closure and restoration of skin barrier function.

### Other purposes

**Figure 5:**
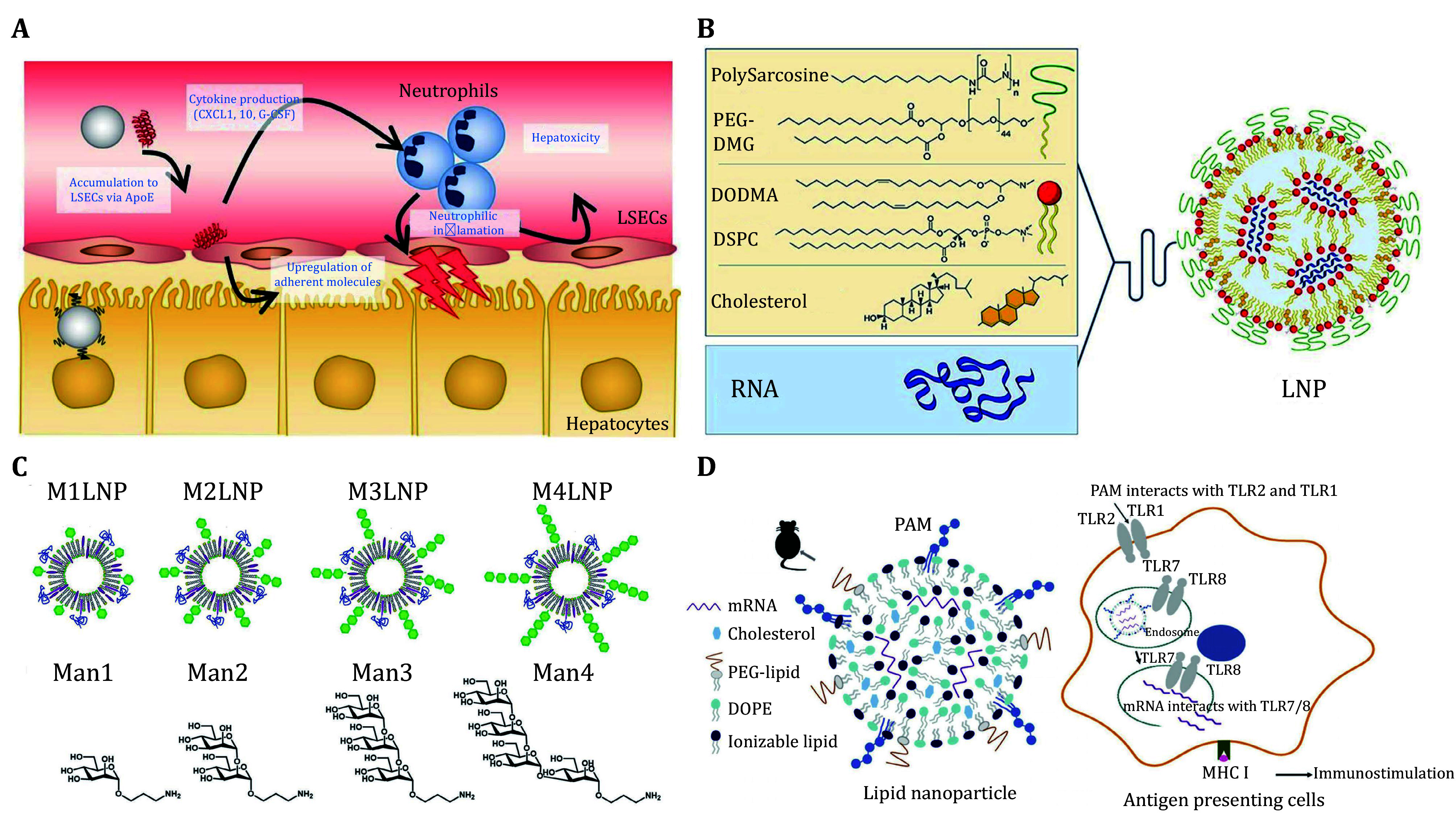
Surface-engineered LNPs for other purposes beyond targeted delivery. **A** Schematic illustration of highly specific delivery of siRNA to hepatocytes, which circumvents endothelial cell-mediated lipid nanoparticle-associated toxicity. Adapted from Sato *et al.* (2017) with permission. **B** Lipid composition used for pSar-LNP manufacturing. Adapted from Nogueira *et al.* (2020) with permission. **C** LNPs modified with mono-(M1), di-(M2), tri-(M3), and tetramannoside (M4) and chemical structure of mannose ligands Man1 (M1), Man2 (M2), Man3 (M3), and Man4 (M4) connected to linker. Adapted from Goswami *et al.* (2021) with permission. **D** Schematic illustration of the Pam3 incorporated LNPs encapsulating mRNA vaccines (Pam-LNPs) for the efficient immune stimulation and subsequent mRNA-mediated cancer immunotherapy. Adapted from Lee *et al.* (2020) with permission

The liver tropism limitation of conventional LNPs substantially drives the development of non-hepatic organ- and specific cell type-targeted LNPs by surface engineering with various targeting ligands. Recently, surface engineering strategies were also utilized to improve the performance and other properties of LNPs beyond the targeted delivery (supplementary Table S3). It was found that LNP accumulation in liver sinusoidal endothelial cells (LSECs) activated endothelial cells and neutrophilic inflammation, which might induce both hepatic and systemic toxicity. To address this issue, LNPs were modified with GalNAc ligands to maximize their hepatocyte specificity while reducing uptake by LSECs (Sato *et al.* 2017). The results showed that systemic administration of GalNAc-LNPs carrying siRNA against hepatitis B virus (HBV) circumvented hepatotoxicity and systemic toxicity in mice with no loss of therapeutic activity in persistent HBV infections (Fig. 5A). To improve the safety profile of LNPs, the “stealth” shell of PEG was replaced by a synthetic non-ionic hydrophilic polypeptoid, pSar_23_ (polysarcosine) (Nogueira *et al.* 2020; Wilhelmy *et al.* 2023). Analysis of *in vivo* systemic toxicity revealed that pSar-LNPs induced similar or lower liver enzyme levels (*e*.*g*., alanine aminotransferase/ALT, aspartate transaminase/AST, lactate dehydrogenase/LDH, and total bilirubin) and reduced cytokine induction compared to their PEG-LNP counterparts (Nogueira *et al.* 2020). Furthermore, a higher and prolonged erythropoietin (EPO) secretion was observed in mice treated with pSar-LNPs containing DPL14 ionizable lipid (Fig. 5B). Enhanced transfection efficiency and cell-binding affinity to monocytes were also achieved with pSar-LNPs *in vitro* (Wilhelmy *et al.* 2023).

Intracellular kinetics of nucleic acid-LNPs can also be affected through the surface engineering. For example, a cY peptide that is composed of a targeting peptide and a cationic domain (16 lysines) linked by a cleavable RVRR linker was surface decorated on LNPs, and it was found that the cleavage of RVRR in the endosomes could trigger the disengagement of LNPs from the targeted receptors, facilitating the endosomal release of the cargoes (Sanghani *et al.* 2021). In another study, a microtubule-associated nuclear localization peptide (MTAS-NLS)-conjugated lipid was incorporated into anti-CD3-LNPs to increase the transfection efficiency of plasmid DNA encoding CD19 CAR in T cells (Zhou *et al.* 2022). The added MTAS-NLS peptide adsorbed the plasmid and provided an enhancement of its condensation, which promoted the nucleus localization of the plasmid and improved the expression of the CAR gene *in vivo*.

Another major application of surface engineering is to modulate the adjuvant activity of mRNA-LNP-based vaccines. Previous studies have demonstrated that mannosylated LNPs enabled enhanced delivery of mRNA to immature DCs and macrophages, which resulted in improved immune responses to vaccines through mannose receptor-mediated LNP endocytosis (Copland *et al.* 2003; Nahar *et al.* 2022; Patil and Deshpande 2020). Recently, studies have shown that mannosylation of self-amplifying RNA-LNPs could not only produce higher antibody titers but also induce a more rapid onset of the immune response compared to unmodified LNPs by both intramuscular (IM) and intradermal (ID) administration routes *in vivo* (Goswami *et al.* 2019, 2021) (Fig. 5C). In addition to mannose, Lee *et al.* have shown that the incorporation of additional adjuvants into LNPs could further boost the mRNA-mediated immune responses (Lee *et al.* 2020). A well-known adjuvant lipopeptide Pam3 that recognizes Toll-like receptor (TLR) 2 and 1 existing on cellular membranes was surface modified on tumor antigen-encoded mRNA-LNPs, and the results showed that stimulation of TLR 2 and 1 significantly enhanced cellular immune responses and produced a high population of antigen-specific CD8+ T cells, which resulted in improved inhibition of tumor growth in tumor-bearing mice (Fig. 5D). Besides, an α-helical cationic peptide KALA (amino acid sequence: WEAKLAKALAKALAKHLAKALAKALKA) was also used to functionalize LNPs for increasing the potency of an *ex vivo* dendritic cell-based cancer vaccine (Tateshita *et al.* 2019). The results showed that KALA-LNPs were able to mediate higher antigen-presentation activity and induce enhanced anti-tumor effects *in vivo* by both efficient cytoplasmic delivery of mRNA and augmented expression *via* avoiding the activation of pathways that down-regulate mRNA translation.

## SUMMARY AND PERSPECTIVES

Since the approval of the first siRNA drug, patisiran, for the treatment of hATTR and the recent two mRNA vaccines against COVID-19, tremendous efforts have been made to develop novel gene therapeutics based on lipid nanoparticle technology. However, limited by the liver accumulation of LNPs *in vivo*, the extension of clinical applications in treating non-liver-related diseases has proceeded slowly so far. The efficient and specific delivery of nucleic acids to other organs is, *per se*, becoming a key challenge in this research field. As the organ tropism of LNPs is highly dependent on their surface properties, engineers and scientists are seeking to construct organ- or cell-type-targeted LNPs by surface engineering. In this review, we first describe the current strategies for incorporating various types of ligands into the LNP surface, and then summarize the purification and characterization methods following the modification step. In the end, we outline the organs and cell types that are currently accessible by the surface-engineered LNPs and also discuss the other purposes of fabricating these LNPs beyond the targeted delivery.

For active targeting approaches, the selection of an appropriate receptor that is expressed in the targeted tissues or specific cell types is particularly important. It was reported that when different receptors (CD117, CD49d, CD44, and IL-6R) were screened for targeting hematopoietic stem and progenitor cells (HSPCs), only the CD117 receptor was effective for LNP uptake and nucleic acid delivery (Shi *et al.* 2023). Generally, the ideal targeted receptor is the one that is highly expressed on cell membranes, can bind to its ligand with high affinity and specificity, enables rapid and efficient internalization, allows for fast receptor recycling, and has controllable biological responses upon binding. When a targeted receptor is defined, different types of ligands, including small molecules, peptides, aptamers, polymers, and monoclonal antibodies, can be chosen to realize the binding purpose. Small molecules and peptides are considered to be easier to manufacture than aptamers and antibodies, while aptamer- and antibody-based ligands generally have stronger binding affinity and a higher degree of cell specificity. Regarding antibodies that are often partially reduced or thiolated for conjugation, the binding affinity before and after modification should be carefully investigated. The study has shown that chemically conjugated antibodies caused a 4.5-fold loss in activity compared to antibodies decorated using the ASSET platform, whose binding is driven by affinity (Kedmi *et al.* 2018). Ligand density is another parameter that requires fine tuning to maximize activity. On one hand, low ligand density could provide insufficient binding to the receptors and induce inefficient uptake by targeted cells; on the other hand, high ligand density might impair the binding due to the steric hindrance and reduce the endosomal escape of payloads. In addition, the potential off-target effects may occur when other tissues or cell types have a low-level expression of the targeted receptor.

Once the targeting ligand is determined, different incorporation strategies need to be tested. For those targeting moieties that are well tolerated in organic solvents, either the post-modification/insertion or the direct “in-lipid mixing” method is feasible. Both strategies have been proven to allow for the formulation of targeted nanoparticles that are able to successfully recognize the corresponding receptor (Valetti *et al.* 2014). Although the “in-lipid mixing” method might make part of targeting moieties be entrapped in the inner core of the nanoparticles during the self-assembly process, it was found that the nanoparticles generated by this method possessed enhanced target binding and better specific avidity compared to those nanoparticles prepared by the post-modification (Valetti *et al.* 2014). Moreover, the “in-lipid mixing” method enables the generation of more stable and uniform nanoparticles than the post-insertion method. For instance, a population of post-insertion prepared LNPs that did not contain GalNAc-lipid was observed by a lectin binding assay (Kasiewicz *et al.* 2023). Besides, the effect of serum proteins on ligand-modified LNPs cannot be ignored. Although it is clear that serum proteins will be adsorbed onto the surface of LNPs following the desorption of PEG-lipids upon systemic injections, the protein corona of different ligand-modified LNPs is not well studied yet. A recent study has reported that the nanoparticle protein corona would achieve a maximum thickness in biological fluids when the protein adsorption and desorption rates reached an equilibrium (Stordy *et al.* 2022). And conjugating targeting ligands to the equilibrated protein corona of the nanoparticles enabled efficient delivery to target cells, while conjugating the ligands to the naked nanoparticles resulted in a 55% reduction of binding affinity to target cells in serum, indicating the potential negative impact of serum proteins on ligand-modified nanoparticles.

While various types of ligands were incorporated into LNPs to improve the specific delivery of nucleic acids, the major accessible organs are still limited to the liver, lungs, spleen, and tumor tissues. Indeed, organ-specific delivery is often dependent on passive distribution. Only when LNPs reach the organ of interest can they make full use of their targeting moieties to realize specific binding and enhanced uptake (Kedmi *et al.* 2018). Therefore, it is not surprising that the liver, lungs, and spleen, where LNPs distribute most, and tumors whose vascularization promotes LNP accumulation, are generally easier to reach than other organs. In our view, future directions in LNP-based targeted nucleic acid delivery will be mostly aimed at developing the targeted LNPs to reach the hard-to-access organs (*e.g.*, the brain, kidneys, heart, eyes, and bone) and cell types. A recent study has shown that intraperitoneal administration of LNPs containing cationic helper lipids induced efficient and specific mRNA delivery in the pancreas, which was mediated by peritoneal macrophage exosome secretion (Melamed *et al.* 2023). Another study has demonstrated that intralesional administration of hIL-10 (human interleukin-10) mRNA-LNP could result in a significant reduction of the microglia/macrophage reaction in the injured spinal segment, which achieved improved functional recovery in rats (Gál *et al.* 2023). These findings give us an inspiration that combining the active targeting ligands with different administration routes may serve as a promising strategy to target the currently inaccessible organs. Besides, LNPs still face other long-standing problems that restrict their broad applications in the clinic, including poor endosomal escape, potential immunogenicity and toxicity, and strict storage conditions. We expect to see more potent, safer, and more stable LNPs using surface engineering strategies that will generate more novel nucleic acid therapeutics and ease manufacturing as well as storage in the future.

## Conflict of interest

Yi Lin, Qiang Cheng and Tuo Wei declare that they have no conflict of interest.
